# ﻿New stenurothripid thrips from mid-Cretaceous Kachin amber (Thysanoptera, Stenurothripidae)

**DOI:** 10.3897/zookeys.1192.117754

**Published:** 2024-02-21

**Authors:** Dawei Guo, Michael S. Engel, Chungkun Shih, Dong Ren

**Affiliations:** 1 College of Life Sciences, Capital Normal University, Beijing 100048, China; 2 Division of Invertebrate Zoology, American Museum of Natural History, Central Park West at 79th Street, New York, NY 10024-5192, USA; 3 Facultad de Ciencias Biológicas, Universidad Nacional Mayor de San Marcos, Lima, Peru; 4 Departamento de Entomología, Museo de Historia Natural, Universidad Nacional Mayor de San Marcos, Av. Arenales 1256 Jesús María, Lima, Peru; 5 Department of Paleobiology, National Museum of Natural History, Smithsonian Institution, Washington, DC 20013-7012, USA

**Keywords:** Cenomanian, new genus, new species, pollinating insects, taxonomy, Thysanoptera

## Abstract

Hitherto, only two species of the thysanopteran suborder Terebrantia have been reported from mid-Cretaceous Kachin amber (Myanmar). This is here expanded through the discovery of two new genera and species, described and figured as *Parallelothripsseparatus***gen. et sp. nov.** and *Didymothripsabdominalis***gen. et sp. nov.**, both of the family Stenurothripidae. Both taxa have key apomorphies of the Stenurothripidae, allowing for a confident assignment as to family. Both species have characteristic comb-like anteromarginal setae, which are discussed along with structural differences between the two sexes. Cycad pollen was found on the thrips’ bodies, providing further evidence that Thysanoptera were pollinators of gymnosperms during the mid-Cretaceous.

## ﻿Introduction

Thrips, order Thysanoptera, comprise a group of small paraneopteran insects with piercing asymmetrical mouthparts, often a characteristic pretarsal bladder, and bearing simplified, linear wings with reduced or absent venation and fringe cilia on the margins. The right mandible of thrips was lost in their evolution, with the left mandible forming a unique asymmetrical feeding tube with the maxillary stylets. The order comprises more than 6600 species, classified into 14 families and 857 genera, with 187 species and 70 genera known only from the fossil record (ThripsWiki 2023).

Thysanoptera is divided into two extant suborders: the reciprocally monophyletic Terebrantia Haliday, 1836 and Tubulifera Haliday, 1836 (Grimaldi and Engel 2005; Buckman et al. 2013; Johnson et al. 2018). These clades are principally distinguished by the form of the tenth abdominal segment, but also by their wing structure (Ulitzka 2022), behavior and development. Tubulifera, as their name suggests, have an elongate, tubular tenth segment and lay eggs on the plant’s surface, while Terebrantia have a sawlike ovipositor and lay eggs within plant tissue. In addition, Tubulifera have three “pupal” stages, while Terebrantia have two.

Some thrips are important pollinators, and occupied such a role even prior to the rise of angiosperms (Willmer 2011). The earliest record of thrips pollination is from Albian amber, approximately 110–105 million years ago (Peñalver et al. 2012). Thrips feed on pollen or other plant tissues and lay eggs within or on the same plants, and their larvae also feed on the flowers (Bailey 1949); in many species adults transport pollen between plants, thereby completing the plants’ reproduction. Today thrips pollination can lead to higher fruiting success in many plants (Garcia-Fayos and Goldarazena 2008; Eliyahu et al. 2015), and instances of thrips-host plant coevolution have been documented (Brookes et al. 2015). Notable examples of thrips pollination include cycads that attract small pollinators such as thrips and weevils, which can enter their ovulate cones while larger insects are excluded (Terry 2001). Indeed, some flowers emit different odors or regulate their temperature to either attract thrips or encourage them to depart from the plant (Terry et al. 2007).

Up to now, species of Thysanoptera recorded from the Cretaceous include the families of Aeolothripidae, Melanthripidae, Merothripidae, Rohrthripidae, Stenurothripidae and Thripidae, and nearly all of these are based on specimens included in amber and more than half of those from the mid-Cretaceous of Kachin, Myanmar (Table 1). Among fossil insect species reported from Kachin amber, the orders Coleoptera (532 species), Hymenoptera (344 species), Diptera (253 species), and Hemiptera (224 species) account for the greatest numbers (Ross 2023), while only 19 species in seven genera from three families have been reported for Thysanoptera (Ulitzka 2018, 2019, 2022; Tong et al. 2019).

**Table 1.** Checklist of thrips reported from Cretaceous amber, with locality and reference indicated.

Suborder	Family	Genus species	Locality	Reference
Tubulifera	†Rohrthripidae	†*Rohrthripsburmiticus*	Kachin amber	Ulitzka 2018
		†*Rohrthripslibanicus*	Lebanese amber	Nel et al. 2010
		†*Rohrthripsbreviceps*	Kachin amber	Ulitzka 2019
		†*Rohrthripsjiewenae*	Kachin amber	Ulitzka 2019
		†*Rohrthripsmaryae*	Kachin amber	Ulitzka 2019
		†*Rohrthripsschizovenatus*	Kachin amber	Ulitzka 2019
		†*Rohrthripspatrickmuelleri*	Kachin amber	Ulitzka 2019
		†*Rohrthripsbrachyvenis*	Kachin amber	Ulitzka 2022
		†*Rohrthripsmultihamuli*	Kachin amber	Ulitzka 2022
		†*Rohrthripspandemicus*	Kachin amber	Ulitzka 2022
		†*Rohrthripsrhamphorhynchus*	Kachin amber	Ulitzka 2022
		†*Rohrthripssetiger*	Kachin amber	Ulitzka 2022
		†*Sesquithripsmarkpankowskii*	Kachin amber	Ulitzka 2022
		†*Sesquithripsrostratus*	Kachin amber	Ulitzka 2022
		†*Adstrictubothripsmirapterus*	Kachin amber	Ulitzka 2022
		†*Gemineurothripsmicrocephalus*	Kachin amber	Ulitzka 2022
		†*Gemineurothripspeculiaris*	Kachin amber	Ulitzka 2022
		†*Paralleloalathripsbivenatus*	Kachin amber	Ulitzka 2022
Terebrantia	Aeolothripidae	†*Cretothripsantiquus*	New Jersey amber	Grimaldi et al. 2004
	Melanthripidae	†*Gymnopollisthripsmaior*	Spanish amber	Peñalver et al. 2012
		†*Gymnopollisthripsminor*	Spanish amber	Peñalver et al. 2012
	Merothripidae	†*Jezzinothripscretacicus*	Lebanese amber	zur Strassen 1973
		†*Myanmarothripspankowskiorum*	Kachin amber	Ulitzka 2018
	Thripidae	†*Tethysthripshispanicus*	Spanish amber	Nel et al. 2010
		†*Tethysthripslibanicus*	Lebanese amber	Nel et al. 2010
	Stenurothripidae	†*Cenomanithripsprimus*	Kachin amber	Tong et al. 2019
		†*Exitelothripsmesozoicus*	Lebanese amber	zur Strassen 1973
		†*Neocomothripshennigianus*	Lebanese amber	zur Strassen 1973
		†*Progonothripshorridus*	Lebanese amber	zur Strassen 1973
		†*Rhetinothripselegans*	Lebanese amber	zur Strassen 1973
		†*Scaphothripsantennatus*	Lebanese amber	zur Strassen 1973
		†*Scudderothripssucinus*	Lebanese amber	zur Strassen 1973
		†*Hispanothripsutrillensis*	Spanish amber	Peñalver and Nel 2010

Stenurothripidae Bagnall, 1923 are a rather small family of Terebrantia, which includes six extant species and 19 extinct species, among which about half of the species were found in Baltic amber (ThripsWiki 2023). The most significant feature of this family is the antenna with nine antennomeres and a broad-based conical sensorium on antennomeres III and IV (Peñalver and Nel 2010; Nel et al. 2012). Extant genera of this family were once placed in Adiheterothripidae (Bhatti 2006), but the family was subsequently resurrected and removed from synonymy (Peñalver and Nel 2010).

Herein we document two new genera and species of Stenurothripidae, *Parallelothripsseparatus* and *Didymothripsabdominalis*, from mid-Cretaceous Kachin amber, enriching the number of species of Thysanoptera from the fossil record. We also discuss their potential interactions with cycads, enlarging the available evidence of gymnosperm pollination by Thysanoptera during the Cretaceous.

## ﻿Materials and methods

The amber fossils studied here were collected from the state of Kachin (Hukawng Valley) of northern Myanmar, located at 26°21'33.41"N, 96°43'11.88"E (Guo et al. 2017). Previous studies have recovered an earliest Cenomanian age for the deposit, approximately 98.79±0.62 Ma (Shi et al. 2012). The amber specimens mentioned in this study were acquired by Mr. Fangyuan Xia before 2015 and donated to us in 2016; both are deposited in the Key Lab of Insect Evolution and Environmental Changes, College of Life Sciences, Capital Normal University, Beijing, China (CNUB, Curator: Dong Ren). The amber specimen is labeled with year of acquisition (e.g., 2016) and specimen accession number (e.g., 116) following the prefix “CNU-THY-MA”.

So as to obtain a better view of specimens, the amber pieces were prepared through a series of steps: initially cut using a razor blade, followed by grinding with Emery papers of different grain sizes, and finally polished with polishing powder. For the current pieces, we produced thin slices, with a thickness of no more than 2 mm.

Specimens were examined and photographed using a Nikon SMZ25 microscope with a Nikon DS-Ri2 digital camera system, illuminated by two or more white-light-LED incident illuminators. White papers were employed as diffusers to prevent reflections on the amber’s surface, and papers with different colors were put under each piece along with transmitting light to create a stronger contrast with the inclusions (as described by Ulitzka 2015). Source images were stacked in Helicon Focus 8 software. Line drawings were prepared with Adobe Illustrator CC 2022 and Adobe Photoshop CC 2020 software.

## ﻿Systematic paleontology

**Family Stenurothripidae Bagnall, 1923**

### ﻿ Parallelothrips

Taxon classificationAnimaliaThysanopteraStenurothripidae

Guo, Engel, Shih & Ren gen. nov.

0C5A9F67-5F9D-5F4B-9293-5405383FE3AD

https://zoobank.org/889D330E-E93A-4171-A53A-0A3A3F45B90C

#### Type species.

*Parallelothripsseparatus* Guo, Engel, Shih & Ren, sp. nov.

#### Etymology.

The new generic name is a combination of the Ancient Greek adjective *πᾰρᾰ́λληλος* (*parállēlos*, meaning, “parallel”) and the Ancient Greek noun *θρίψ* (*thrips*, meaning, “woodworm”). The name refers to the two parallel rows of marginal setae on the pronotum. The gender of the name is masculine.

#### Diagnosis.

**Figure 1.**
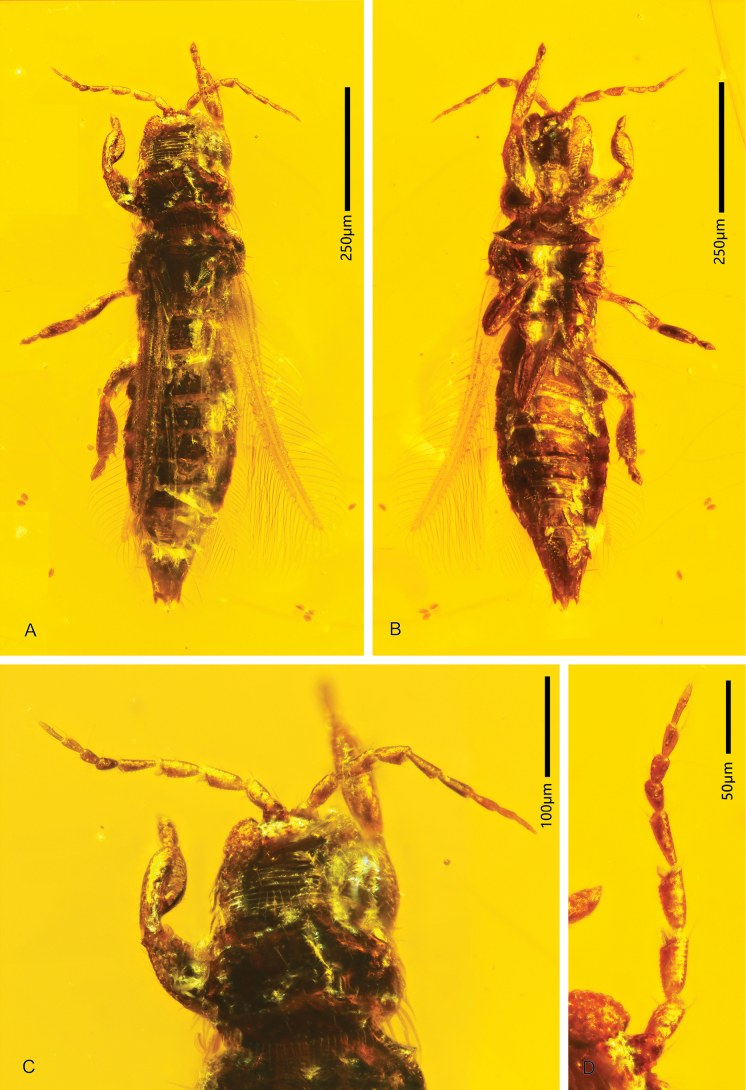
*Parallelothripsseparatus* gen. et sp. nov.; holotype male (CNU-THY-MA2016116) **A** dorsal view **B** ventral view **C** head and pronotum, dorsal view **D** left antenna, conical sensorium present on antennomeres III–IV.

**Figure 2.**
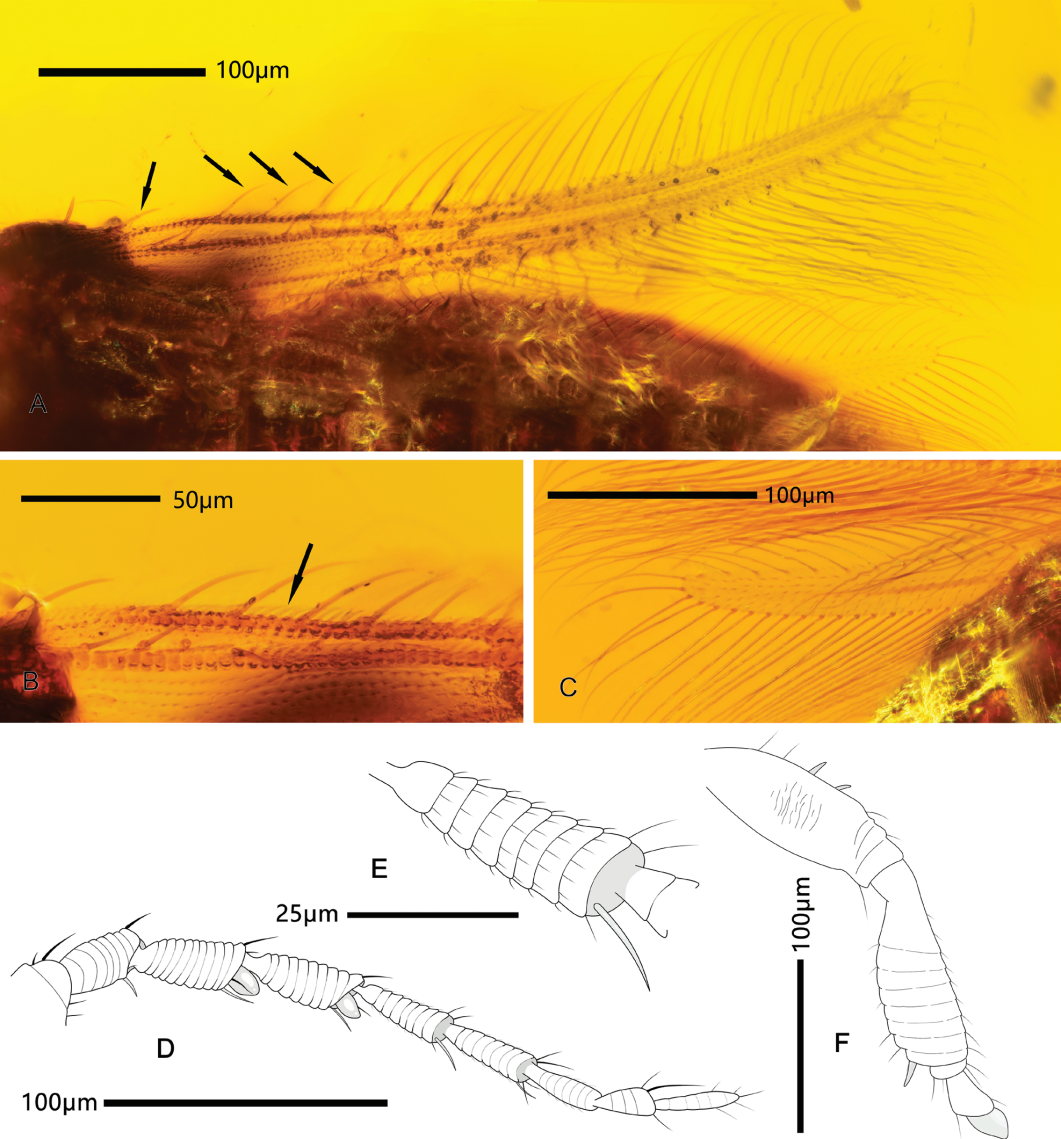
*Parallelothripsseparatus* gen. et sp. nov.; holotype male (CNU-THY-MA2016116) **A** right fore wing, dorsal view, the foremost fringe cilium lacks a corresponding anteromarginal setae (indicated by black arrows) **B** microtrichia of anterior wing margin **C** surface of hind wing, covered with microtrichia **D** ventral view of left antenna **E** antennomere V, showing rounded annulation sculpture and microtrichia **F** dorsal view of hind leg (from metafemur onward).

**Figure 3.**
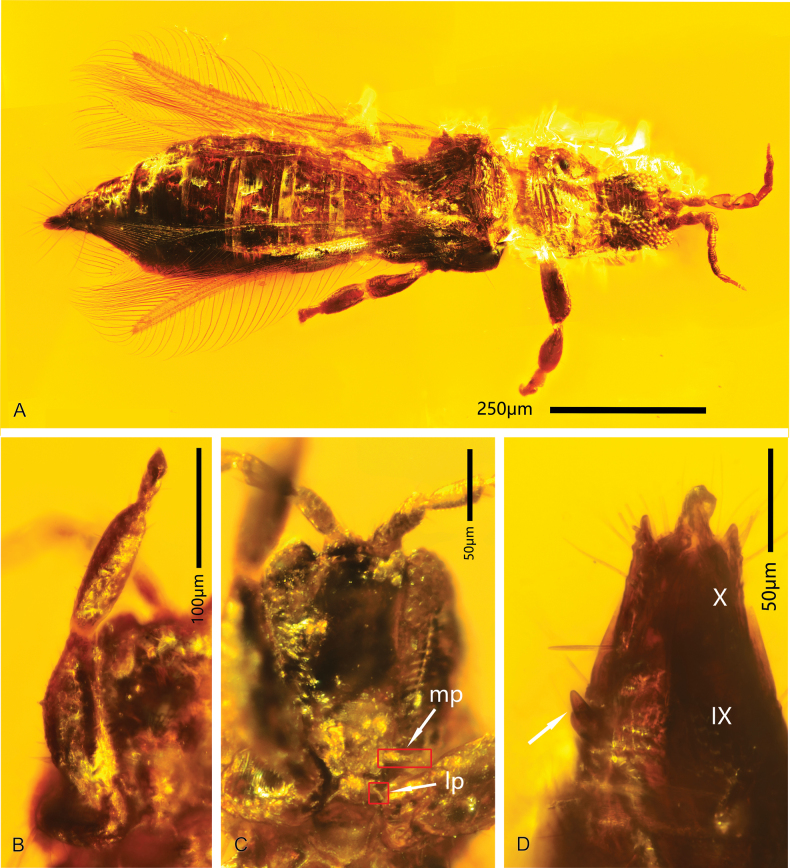
*Parallelothripsseparatus* gen. et sp. nov. **A** female (CNU-THY-MA2016117), dorsal view **B** fore leg of male (CNU-YHY-MA2016116), vesicle cone-shaped at the tip **C** mouth cone (CNU-THY-MA2016116), mp indicated: maxillary palpus, lp indicated: labial palpus **D** distal abdominal segments of male (CNU-THY-MA2016116), spine on segment IX (indicated by a white arrow).

Antenna (Fig. 1) with nine antennomeres, antennomeres III and IV asymmetrical inverse cone-shaped, stouter than distal antennomeres, each with a broad-based conical sensorium. Head (Fig. 1C) dorsally with transverse striations basally, cheek rounded behind compound eye. Compound eye prolonged ventrally. Pronotum wider than head, with striate sculpture basally, studded with regular rows of setae on both anterior and posterior margins, posteroangular setae long and stout. Mesonotum (Fig. 1C) not adjoined to pronotum, with distinct separation between segments. Fore wing (Fig. 2A) narrow, with two longitudinal veins and two crossveins visible, wing surface covered with microtrichia (Fig. 2B), fringe cilia of posterior margin longer than those of anterior margin and slightly undulate, wavy duplicated cilia present around wing tip. Hind wing (Fig. 2C) nearly transparent, surface covered with microtrichia, with one distinct longitudinal vein. Abdomen 10-segmented, about as wide as thorax at widest point, with some strong setae near apex; female segments VIII–X coniform, male (Fig. 3D) segment X rounded.

### ﻿ Parallelothrips separatus

Taxon classificationAnimaliaThysanopteraStenurothripidae

Guo, Engel, Shih & Ren sp. nov.

9ED5768B-CE2B-5F20-9D0C-0EFA25C94D15

https://zoobank.org/B65E3843-59B7-48F1-9757-82F56AD5E1C6

Figs 1 , 2 , 3

#### Type materials.

***Holotype*** male (CNU-THY-MA2016116) and ***paratype*** female (CNU-THY-MA2016117), both as inclusions in Kachin amber piece CNU006122.

#### Etymology.

The specific epithet is the Latin participle *sēparātus*, meaning “divided” or “separated” and referring to the distance between the pronotum and mesonotum of the holotype.

#### Diagnosis.

As for the genus (*vide supra*).

#### Description.

**Holotype male** (CNU-THY-MA2016116). Body, legs, as well as antennae and wing veins uniformly dark brown, right compound eye and wings partly hidden by a shiny reflective layer of air. Antenna (Fig. 1A, C) curved laterally; body fully extended, right forewing spread, some fringe cilia from anterior margin fractured (Fig. 2A); legs (Fig. 1B) extended except right mid and hind legs folded under body.

Head (Fig. 1C) wider than long, dorsally sculptured with transverse striations at base, cheeks rounded behind compound eyes. Compound eye (Fig. 1C) large, with dozens of large ommatidia, front margin protruding over base of antenna, postocular setae as well as ocellar setae short and pointed, directing backward; median ocellus directed forwards, lateral ocelli close to compound eyes. Antenna (Figs 1D, 2D) with nine antennomeres, antennomeres II–IX rounded with transversal annulation, furnished with several microtrichia (Fig. 2E); antennomeres I–IV stouter than distal antennomeres; I broadened basally, III–IV symmetrical inverse cone-shaped and both having a conical sensorium with a broad base; sense cone simple, at least one inner on antennomeres II–VI. Mouth cone (Fig. 3C) short; maxillary palps stout, trimerous; labial palps short and slender.

Pronotum (Fig. 1C) wider than long, dorsally sculptured with transverse striations, lateral margin rounded, tightly adjoined to head; anteromarginal and posteromarginal setae comb-like; anteroangular setae not visible, posteroangular setae long and stout. Mesonotum fan-shaped; sculptured with transverse striations, slightly separated from pronotum. Metanotum sculptured with light longitudinal striations. Fore wing (Fig. 2A) narrow, slightly wider at basal third, distally slightly bent forwards. Wing venation pale in color, anterior vein ending at apex, with seven small setae; posterior vein with six setae distributed evenly, ending near wing tip, one crossvein (r-m) present between them, other above it; membrane (Fig. 2B) covered with microtrichia. Fringe cilia fine and long, those located near duplicated cilia slightly undulate; wavy duplicated cilia present near apex and apical part of posterior wing margin, stouter than other cilia. Anteromarginal setae long and fine, running parallel to direction of fringe cilia and spaced in pairs; foremost fringe cilia lack paired anteromarginal setae, and position of first fringe cilium quiet distant, near base of wing (Fig. 2A). Some microtrichia densely arranged along anterior wing margin, extending from base to apex (Fig. 2B). Clavus with a pair of setiform processes at tip. Hind wing (Fig. 2C) almost transparent, membrane covered with microtrichia, with one longitudinal vein ending nearly at wing apex; fringe cilia from posterior margin longer than that of anterior margin, and both straight. Legs (Figs 2F, 3B) furnished with many microtrichia, hind leg (Fig. 2F) with two spines on femora and a stout spine at the end of tibiae, tarsi dimerous, no hamus present, vesicles cone-shaped.

Abdomen with ten segments, dorsally sculptured with lines of transverse striations; some small setae present on posterior margin of each segment; segment I partly hidden by metanotum, not narrower than thorax at basal segments, slightly bent upwards at apex; with a pair of spines and a pair of short stout setae on segment IX (Fig. 3D); segment X round, pleurite protrudes on both sides, aedeagus protrudes medioapically.

**Paratype female** (CNU-THY-MA2016117, Fig. 3A). Body, antennae, and legs uniformly dark brown, wing surface as well as veins and fringe cilia light brown. Body slightly inclined to left, antennae curved to both sides, pronotum bent downwards, abdomen extended; wings spread; right fore and mid legs spread, other legs bent under head and body.

Nearly identical to male (CNU-THY-MA2016116) in size, but lighter in color. Compound eye protruding over front margin of head, ocellus large. Forewing narrow, duplicated cilia extending from mid-wing to tip, clearly undulate near apex. Fore legs with femora stout, mid and hind legs slender; vesicles rounded at apex of each tarsus. Abdomen slenderer than thorax and gradually broadening before segment VI; latero-tergites protruding on segments III–VI; segments VIII–X cone-shaped, curved downwards, narrow and elongate; surrounded with several long and strong setae apically.

#### Measurements.

Male CNU-THY-MA2016116 (in microns): Body length 946 (antennae not included). Head, length 108; width 155. Eye, length 43; width 44. Prothorax, length 74; width 203. Anteromarginal setae, length 17; posteromarginal setae, length 24; posteroangular setae, length 54. Pterothorax, length 146; largest width 215. Abdomen, length 548; largest width 205 (segment V). Antenna, length 285; lengths of segments: I 20, II 38, III 43, IV 44, V 33, VI 37, VII 25 VIII 16, IX 29. Forewing, length 555, width 40 at crossvein. Hind wing, length 554, largest width 29. Fore leg, length 341; mid leg, length 336; hind leg, length 340.

Female CNU-THY-MA2016117 (in microns): Body length 1010 (antennae not included). Head, length 110; width 122. Eyes, length 61; width 49. Hind ocelli, diameter 8. Prothorax, length 110; width 161. Anteromarginal setae, length 19; posteromarginal setae, length 19; posteroangular setae, length 61. Pterothorax, length 206; largest width 222. Abdomen, length 553; largest width 231 (segment V). Antenna, length 236; lengths of segments: I 22, II 32, III 43, IV 34, V 30, VI 22, VII 14, VIII 13, IX 26. Forewing, length 580, width 31 at crossvein. Hind wing, length 599, largest width 26. Fore leg, length 254.

#### Syninclusions.

**Figure 4.**
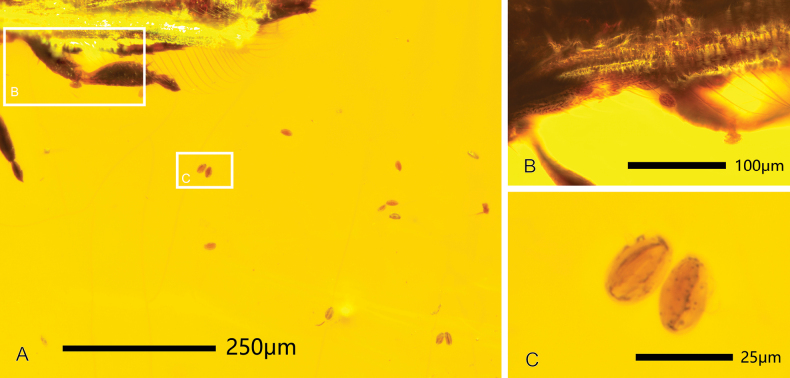
Cycad pollen found in the CNU-THY-MA2016116 **A** pollen distributed around the thrips **B** pollen attached on the fringe cilia and legs **C** enlarged details of the cycad pollen.

Some cycad pollen (Fig. 4) was found around and on the body of the male.

### ﻿ Didymothrips

Taxon classificationAnimaliaThysanopteraStenurothripidae

Guo, Engel, Shih & Ren gen. nov.

E7B090A3-4B63-5589-AA7E-4FAF6F3487C1

https://zoobank.org/C96F2CBF-450D-414F-9E91-AE4CCF232309

#### Type species.

*Didymothripsabdominalis* Guo, Engel, Shih & Ren, sp. nov.

#### Etymology.

The new generic name is a combination of the Ancient Greek *Δίδυμοι* (*Dídymoi*, the original Greek name for the later Roman Gemini) and the Ancient Greek noun *θρίψ* (*thrips*, meaning, “woodworm”). The name refers to the anteromarginal and posteromarginal setae of the pronotum. The gender of the name is masculine.

#### Diagnosis.

**Figure 5.**
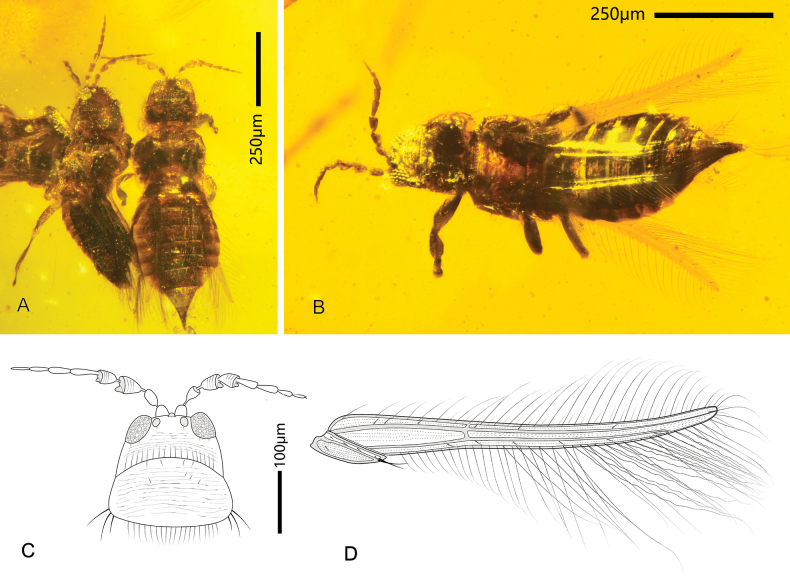
*Didymothripsabdominalis* gen. et sp. nov. **A** holotype female (right) (CNU-THY-MA2016102/1) and paratype male (left) (CNU-THY-MA2016102/2), dorsal view **B** paratype female (CNU-THY-MA2016118), dorsal view **C** head, antenna and pronotum of CNU-THY-MA2016102/1 **D** forewing of CNU-THY-MA2016102/1.

Antenna (Fig. 5A) with nine antennomeres, cone-shaped sensorium present on inverted-triangle shaped antennomeres III–VI. Head (Fig. 5C) dorsally sculptured with transverse striations; compound eye protruding in front of the front margin. Pronotum (Fig. 5C) about as wide as the head, sculptured with transverse striate, furnished with some sparse microtrichia; anteromarginal and posteromarginal setae of pronotum long and comb-like, two pairs of long posteroangular setae present. Mesonotum partly hidden by pronotum and its posteromarginal setae. Forewing (Fig. 5D) narrow, slightly bent forwards and membrane covered with microtrichia; two parallel longitudinal wing veins furnished with several stout setae, crossveins not developed. Fringe cilia long and undulate, duplicated cilia present at distal part of posterior wing margin. Abdomen 10-segmented, showing differences between the male and the female; female (Fig. 5A, B) generally with a flat and wide abdomen with pointed terminal direct backward or slightly downward, while male (Fig. 5A) with a short and thick abdomen with terminal not pointed.

### ﻿ Didymothrips abdominalis

Taxon classificationAnimaliaThysanopteraStenurothripidae

Guo, Engel, Shih & Ren sp. nov.

7A0C9075-7784-5F5C-BAA1-49F955C6769F

https://zoobank.org/B9768AC0-4906-4E12-A555-2C3F6A0712EB

Fig. 5

#### Type materials.

***Holotype*** female (CNU-THY-MA2016102/1) and ***paratype*** male CNU-THY-MA2016102/2 (Fig. 5A), inclusions in Kachin amber piece CNU009269; as well as a paratype female CNU-THY-MA2016118 (Fig. 5B) in Kachin amber piece CNU009461.

#### Etymology.

The specific epithet is from the Latin adjective *abdōminālis*, meaning “abdominal” and referring to the wide abdomen of the species.

#### Diagnosis.

As for the genus (*vide supra*).

#### Description.

**Holotype female** (CNU-THY-MA2016102/1). Body (Fig. 5A) uniformly dark brown, antennae and wing veins light brown, posterior half of pronotum transparent. Antennae (Fig. 5C) bent towards sides, legs spread under body, forewing (Fig. 5A) overlapping body, right hind wing partly covered by forewing, left hind wing almost completely covered by fore wing except apex.

Head (Fig. 5C) wider than long, dorsally sculptured with transverse striations. Cheek nearly straight, slightly diverging posteriorly. A pair of short ocellar setae located behind lateral ocelli; two pairs of postocular setae close to compound eyes. Ocelli rather large, median ocellus near middle of base of antenna, interocellar setae long and direct upward; lateral ocelli located close to compound eye. Compound eye (Fig. 5C) protruding in front of anterior margin of head, with many large ommatidia. Antenna (Fig. 5C) with nine antennomeres; I inverse funnel-shaped, II oval, III–IV inverted-triangle shaped, V–IX elongate club-shaped, II–IX with a pedicle at base. Conical sensorium present on antennomere III but not visible on IV. Mouth cone difficult to observe.

Pronotum (Fig. 5C) wider than long, trapezoidal, tightly adjoined with head; dorsally with transverse sculpture and some sparse setae; anteromarginal and posteromarginal setae long and comb-like; posteroangular setae long and pointed. Mesonotum (Fig. 5A) triangular; dorsally with some transverse striations, partly covered by pronotum. Metanotum dorsally furnished with some stout setae and longitudinal lines, slightly bent upwards. Forewing (Fig. 5D) narrow, slightly bent forwards at apex; two longitudinal veins light in color and parallel, anterior vein ending at apex, with strong setae; posterior vein reaching 4/5 of wing length; two crossveins visible close to each other between anterior wing margin and longitudinal veins at basal third; membrane furnished with many microtrichia. Fringe cilia straight on anterior margin but undulate and a little longer on posterior margin; duplicated cilia undulate (Fig. 5D). Clavus with pair of setiform processes at tip. Hind wing transparent, one longitudinal vein nearly reaching apex, some microtrichia present at apex of wing. Legs long and slender, femora of fore legs slightly enlarged, protarsus without a hamus.

Abdomen (Fig. 5A) flat and broad, dorsally sculptured with transverse striations; abdominal tergite I hidden by metanotum, wider than thorax at middle part, widest at segments V–VI, rapidly tapering in width along segments VIII–X; segment X conically shaped, surrounded with some long straight setae, directed posteriorly.

**Paratype male** (CNU-THY-MA2016102/2, Fig. 5A). Body, legs, and antennae uniformly dark brown, fore wing membrane light brown. Body not fully extended, with head slightly bent downwards and abdomen bent upwards; fore and mid legs recurved under body, blocked by a female thrips (CNU-THY-MA2016102/1), hind legs fully extended; wings overlapping body.

Similar to female in color and most body structures, but smaller and much slender in size. Antenna stouter than female; abdomen short and thick, generally slightly bent upwards, not wider than thorax, edges between different segments distinct, somewhat conical process present on segment IX, segment X round, with two pleurites connected by membrane.

#### Measurements.

Female CNU-THY-MA2016102/1 (in microns): Body length 799 (antenna not included). Head, length 72; width 166. Eye, length 50; width 33. Hind ocelli, diameter 12; distance between the hind ocelli 43. Prothorax, length 134; width 168. Anteromarginal setae, length 24; posteromarginal setae, length 26. Pterothorax, length 169; largest width 250. Abdomen, length 567; largest width 308 (segment V). Antenna, length 262; lengths of segments: I 17, II 33, III 35, IV 37, V 37, VI 33, VII 23, VIII 21, IX 26. Forewing, length 562, width 33 at crossvein. Hind wing, length 551.

Male CNU-THY-MA2016102/2 (in microns): Body length 745 (antenna not included). Head, length 90; width 138. Eye, length 58; width 48. Hind ocelli, diameter 12; distance between the hind ocelli 32. Ocellar setae, length 34. Postocular setae, length 30. Prothorax, length 125; width 185. Anteromarginal setae, length 24; posteromarginal setae, length 26. Pterothorax, length 148; largest width 226. Abdomen, length 379; largest width 221 (segment V). Antenna, length 266; lengths of segments: I 16, II 38, III 37, IV 33, V 31, VI 34, VII 25, VIII 25, IX 27. Forewing, length 604, width 37 at crossvein.

#### Syninclusions.

168 *Stenurothrips* specimens are preserved in the amber CNU-THY-MA2016102, together with a cicadid (Hemiptera), a nematoceran (Diptera), and an apocritan wasp (Hymenoptera). Two stenurothripids, an unnamed species of Thripidae, and an aphid are contained in CNU-THY-MA2016118.

## ﻿Discussion

Schliephake (1990) characterized Stenurothripidae as: antenna with nine antennomeres, all antennomeres freely connected; conical sensorium with a broad base present on antennomeres III and IV; fore wing covered with microtrichia, two longitudinal veins nearly reaching the tip of the wing margin; usually with three to four crossveins between the longitudinal veins or between them and the marginal vein; fringe cilia wavy (for most fossil species) or straight (for extant species) on the posterior margin of the fore wing; and distal tarsomere with a hook-shaped tooth (hamus) ventrally (Schliephake 1990). The fossil specimens from the three Kachin amber pieces (CNU006122, CNU009269 and CNU009461) share most of the characters listed above except that they lack the tarsal hamus, and only two crossveins in the forewing are visible, but are otherwise most consistent with Stenurothripidae.

The two new genera established here have characteristic comb-like anteromarginal setae (Figs 1C, 5C) on the pronotum, which are yet to be found elsewhere in the family and, therefore, are considered apomorphies of these new genera. In addition, they are distinguished from other stenurothripid genera as follows: *Stenurothrips* have an elongate tenth abdominal segment, which is not the case in the two new genera; the new genera have narrow wings whereas *Exitelothrips*, *Neocomothrips* and *Progonothrips* have broad wings; the genera *Oligothrips* and *Hispanothrips* have a hamus present on the protrasus, which is absent in the two new genera. Note that *Rhetinothrips* and *Progonothrips*, both from Lebanese amber, should perhaps be classified in their own subfamily distinct from other Stenurothripidae.

It is tempting to classify the species here into a single genus with anteromarinal pronotal setae were it not for the considerable differences in their overall morphology. For example, in the female the head of *Parallelothrips* is narrower than the pronotum, while the head of *Didymothrips* is about as wide as the pronotum and they are closely adjoined. The mesonotum of *Parallelothrips* is not closely adjoined to the pronotum, and only the middle part of the anterior margin protrudes forward, whereas they are closer in *Didymothrips* with the mesonotum partly hidden by the pronotum. The legs (Figs 3A, B, 5A) of *Parallelothrips* and *Didymothrips* are significantly different, with the latter thinner and not showing any sexual dimorphism. *Parallelothrips* has a slender abdomen with an elongate cone-shape at the end, only a little wider than the thorax, while *Didymothrips* has a broad and flat abdomen with a stout cone-shape tip, much wider than the thorax. The differences between the males are even more significant; males of *Parallelothrips* are similar to females in body size and shape, except for the last three abdominal segments while the males of *Didymothrips* (Fig. 5A) are obviously smaller than their females, with abdomen more slender and thicker. Significant distinctions in body structures are used here to establish two separate, but likely closely related, genera, enriching the known diversity of thrips from Kachin amber.

As we have males and females of both new species, we were able to document sexual dimorphism for both cases. In *Parallelothripsseparatus*, females and males differ in the form of their legs as well as the shape of the abdominal apex, while in *Didymothripsabdominalis* the differences between the two sexes are mainly reflected in the abdominal form.

All of the thrips currently documented from Kachin amber have nine antennomeres, a condition considered plesiomorphic for crown-Thysanoptera (Mound et al. 1980). According to a phylogenetic estimation based on morphology (Nel et al. 2014), Stenurothripidae and Thripidae are closely related as they share the following characters: both have narrow wings with M emerging from R proximally or near to the RA-RP fork and the ovipositor is straight or downcurved (Nel et al. 2012). The most recognizable feature of Stenurothripidae is the presence of the broad-based conical sensorium on antennomeres III and IV, which cannot be observed in all of our specimens due to preservation but can be sufficiently discerned in enough individuals to indicate they are Stenurothripidae.

Compared to extant Stenurothripidae, the new species have simple wing venation (Figs 2A, 5D) and fewer crossveins; the wavy duplicated cilia on the forewing are present around the margin of the wing apex and extend to the middle of the posterior wing margin. These cilia extend apically, and each forms a pair with a normal fringe; this situation is similar to other thrips documented from mid-Cretaceous Kachin amber inclusions.

The pollen grains (Fig. 4) around the thrips in CNU-THY-MA2016116 are tiny (average 21.0 μm long and 12.6 μm wide) with a spindle to ovoid shape, along with a smooth surface and a groove medially, and decorated with a few small dark spots laterally (likely pits or punctures). The medial groove is as long as the pollen grain and the depth can reach up to half of the thickness, slightly wider at both ends and narrower medially (Fig. 4C). These characteristics suggest that the pollen grains are from the gymnosperm form-genus *Cycadopites*.

The pollination efficiency of thrips depends mainly on two aspects, the ability to carry pollen, which is determined by the number of setae on the body, and the overall mobility of the animal related to the presence or absence of wings (Lewis 1973). In contrast with *Gymnopollisthrips* (Peñalver et al. 2012), there are no specialized ring setae on the new species, while *Parallelothrips* has many setae on its body, legs, and antennae, and a greater number of anteromarginal bristles and duplicated cilia present on the forewing, which could enhance the capture and transport of pollen grains. Indeed, in most of the specimens of *P.separatus* the posterior margin of the pronotum is not closely adjoined to the anterior margin of the mesonotum and some pollen grains were found affixed to the posteromarginal and posteroangular setae extending between the two sides (Fig. 5B), suggesting that this structure was likely specialized for the transport of pollen grains.

Here we document two new genera of Thysanoptera from mid-Cretaceous Kachin amber, expanding the known diversity of the order and the family Stenurothripidae, in particular. Currently, Stenurothripidae have been found in the Cretaceous from Lebanon, Spain, and Myanmar, demonstrating their wide distribution at the time. These genera were likely pollinators of gymnosperms during the mid-Cretaceous.

## Supplementary Material

XML Treatment for Parallelothrips

XML Treatment for Parallelothrips separatus

XML Treatment for Didymothrips

XML Treatment for Didymothrips abdominalis

## ﻿Additional information

### Author ORCIDs

Dawei Guo https://orcid.org/0009-0009-4349-5545

Michael S. Engel https://orcid.org/0000-0003-3067-077X

Chungkun Shih https://orcid.org/0000-0002-3434-2477

Dong Ren https://orcid.org/0000-0001-8660-0901

### Data availability

All of the data that support the findings of this study are available in the main text.
